# Low muscle quality, but not muscle mass, in abdominal CT-scans associates with adverse outcomes in AML patients undergoing an allogeneic stem cell transplantation

**DOI:** 10.1038/s41409-026-03005-w

**Published:** 2026-08-13

**Authors:** Jule Ussmann, Clara Wall, Lara Bischof, Jacob Jendro, Sofia Voigt, Dominic Brauer, Donata Backhaus, Maximilian Merz, Vladan Vučinić, Marco Herling, Carmen Herling, Georg-Nikolaus Franke, Uwe Platzbecker, Timm Denecke, Klaus H. Metzeler, Sebastian Schwind, Hans-Jonas Meyer, Madlen Jentzsch

**Affiliations:** 1 Department for Hematology, Cellular Therapies, Hemostaseology, and Infectious Diseases, University of Leipzig Medical Center, Leipzig, Germany; 2 Cancer Center Central Germany – CCCG, Leipzig, Germany; 3 Department for Diagnostic and Interventional Radiology, University of Leipzig Medical Center, Leipzig, Germany

**Keywords:** Acute myeloid leukaemia, Medical research, Risk factors

**To the editor**,

For many patients with acute myeloid leukemia (AML), allogeneic hematopoietic stem cell transplantation (HSCT) remains the treatment with the highest curative potential. Non-relapse mortality (NRM) is a relevant competing risk after HSCT, with a cumulative risk affecting up to 49% of patients after 3 years [1]. Patient-specific and—to some extent—modifiable factors to potentially reduce NRM are lacking.

Recently, computed tomography (CT)-defined body composition parameters emerged as patient-related risk factors for NRM in solid cancers [2, 3]. With muscle mass and muscle quality, two surrogate parameters for sarcopenia have been recognized and are measured in CT on the level of the third lumbar vertebra (L3) as the current gold standard (Fig. S1) [4]. In solid neoplasms, both low muscle mass and low muscle quality have been associated with shorter overall survival (OS) and increased treatment-related toxicity [3, 4]. Similar results were found in previous studies in unselected cohorts of patients with hematologic neoplasms treated with HSCT [5, 6]. In AML patients undergoing intensive treatment, treatment-related toxicities increase the risk for weight loss during treatment. While such weight loss between diagnosis and HSCT in AML patients has been associated with inferior outcomes [7], existing data don’t acknowledge which tissue types are accountable for body weight changes.

To further investigate this topic, we retrospectively analyzed 429 AML patients (median age 59 [range 17-76] years; 46% female) who received an HSCT at our center and had an abdominal CT-scan up to 7 weeks prior to HSCT available. Muscle mass was evaluated through skeletal muscle index (SMI, i.e., skeletal muscle area divided by the body height squared). While two-thirds of patients were sarcopenic, defined by muscle mass, a low SMI did not associate with adverse outcomes (Fig.S2). An explanation might be that the established cut-off values were derived in patients with respiratory and gastrointestinal neoplasms [2], which might affect the muscle long before diagnosis. In contrast, AML usually develops quickly and is diagnosed shortly after the first symptoms, leaving less time for significant loss of muscle mass.

One other study associated a lower SMI in 859 patients undergoing HSCT (54% AML) with adverse NRM and OS [6]. However, here, only 34% of patients were sarcopenic, likely a result of different cut-offs used, which were also BMI-adapted in male patients. This shows a weakness of the SMI, as it only quantifies the area of muscle without detecting intramuscular adipose tissue, and thus, may overestimate the lean muscle mass in some patients [8].

To account for a potential muscle infiltration by adipose tissue, which can decrease muscle strength and function without necessarily impacting muscle mass [8], the radiodensity of the muscle can be considered. One previous study showed a lower muscle quality associated with shorter OS and higher NRM in 186 patients with unselected hematologic neoplasms [9]. However, apart from the high heterogeneity of the cohort (only 35% AML), the radiodensity of the muscle alone was used as a surrogate for muscle quality. In contrast, the intramuscular adipose tissue content (IMAC), as described by Hamaguchi et al. [10], represents a more robust parameter. The IMAC is calculated by dividing the mean skeletal muscle radiodensity by the radiodensity of subcutaneous adipose tissue and subsequently reduces alterations through patient-specific deviations in tissue-density as well as differing CT parameters. Thus, we used the IMAC to estimate muscle quality in our study. As no established cut-offs for the IMAC in cancer patients exist, we used receiver operating characteristic (ROC) curves in a test and validation set to define informative sex-specific cut-offs (for further details see Supplementary Material).

In our AML cohort, an IMAC above the sex-specific cut-off represented a strong risk factor for higher NRM (*P* < 0.001) and shorter OS (*P* < 0.001, Fig. 1a, b) after allogeneic HSCT. A higher IMAC was linked to older patient age (*P* < 0.001, Fig. 1c), a higher body mass index (BMI, *P* = 0.001, Fig. 1e), and, subsequently, to a variety of adverse risk characteristics in AML patients undergoing allogeneic HSCT, such as adverse ELN2022 risk (*P* = 0.03), a higher HCT-CI risk score (*P* = 0.02), and less intensive conditioning (*P* < 0.001, for detailed results see Table S1). We additionally observed a higher incidence of acute (*P* = 0.001) and chronic graft-versus-host disease (GvHD, *P* = 0.04), likely as a result of the non-myeloablative conditioning regimen used in older individuals, which did not contain in-vivo T-cell depletion. Still, the IMAC also remained an independent prognostic factor of 3.6-times higher risk of NRM and 2.2 times lower chance for OS in multivariate analyses (Table S2).

**Fig. 1:**
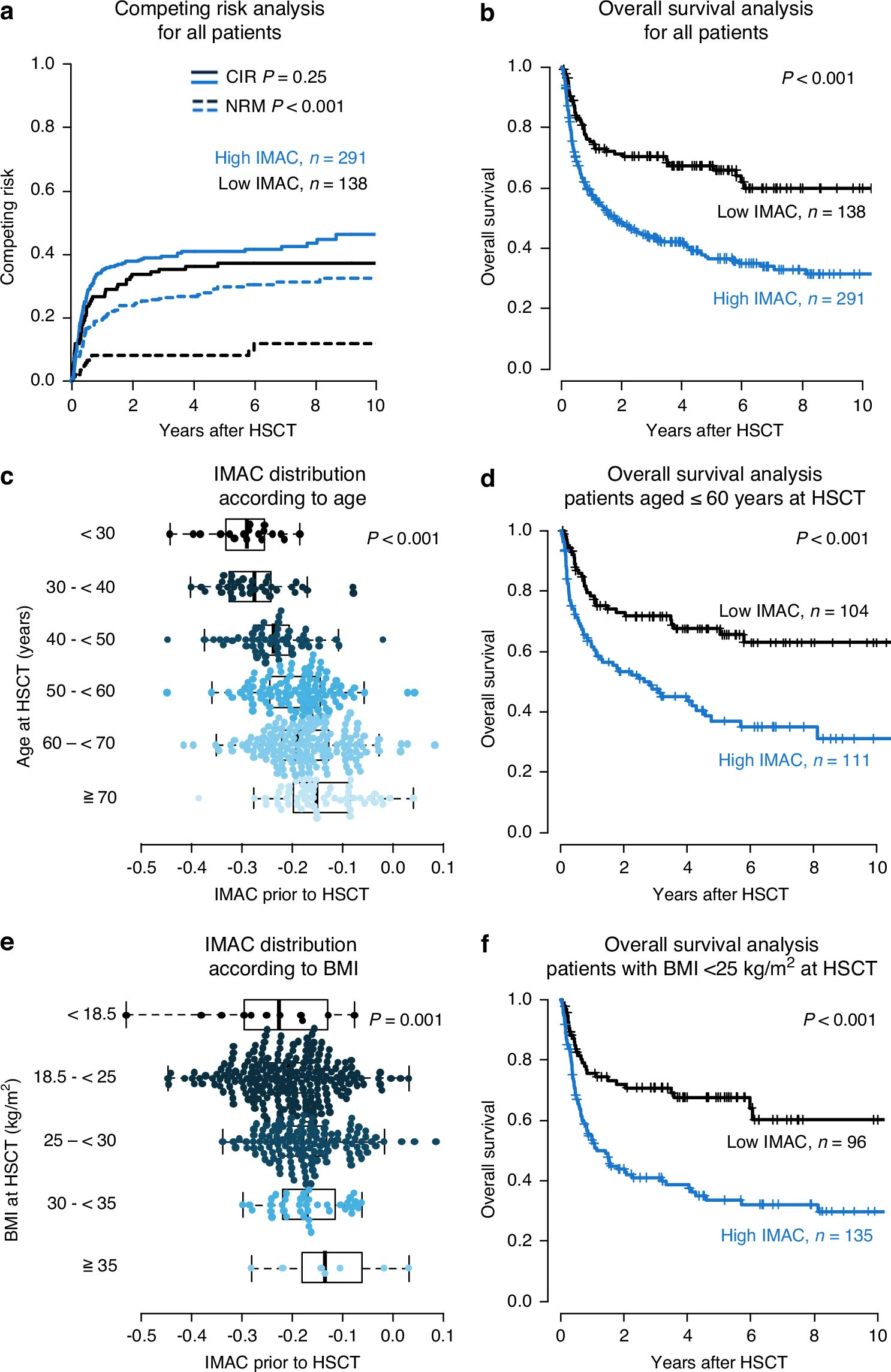
Outcomes of AML patients undergoing allogeneic HSCT according to muscle quality defined by the IMAC prior to HSCT (high *vs* low, optimal cut: males –0.2469; females –0.2146). **a** Competing risk analysis (Cumulative incidence of relapse/progression and non-relapse mortality) and **b** Overall survival of all patients. **c** Display of IMAC distribution according to age at HSCT. **d** Overall survival of patients aged ≤ 60 years at HSCT. **e** Display of IMAC distribution according to the body mass index (BMI) at HSCT. **f** Overall survival of patients with normal or underweight (BMI < 25) at HSCT.

Because of the mentioned relevant correlations, subgroup analyses according to age and BMI at allogeneic HSCT were performed. Here, we observed that the IMAC showed a particularly strong association with OS in patients younger than 60 years at HSCT (*P* < 0.001, Fig. 1d), but less in older individuals (*P* = 0.04, Fig. S4B), as well as in patients of under- or normal weight (*P* < 0.001, Fig. 1f) but not in obese patients (*P* = 0.40, Fig. S4D). This reflects a population of AML patients that is not usually suspected to present with sarcopenia, but that might respond well to interventions to preserve skeletal muscle mass.

Currently, transplant physicians assess patient eligibility for HSCT as well as the conditioning intensity mainly based on the patient’s age and comorbidity burden. While the performance status remains important, a patient’s overall fitness is often evaluated based on subjective estimation and personal experience. Here, CT-based muscle parameters could help to more objectively detect patients at higher risk of NRM, especially in young and normal-weight individuals, who are often perceived as rather fit. This is particularly relevant in individuals who received CT-scans within the clinical routine to rule out pathologies prior to HSCT, where the IMAC can be assessed as a promising additional risk factor without additional exposure to radiation.

First attempts to assess body composition and physical functioning, as well as to maintain muscle strength during allogeneic or autologous HSCT, have been conducted [11, 12], and some already show positive effects of strength and endurance training and/or protein supplementation. However, the dynamics of sarcopenia as well as factors influencing muscle mass and quality prior to allogeneic HSCT remain to be further investigated.

To our knowledge, this is the first study that focuses on the impact of muscle mass and quality in a large cohort of AML patients undergoing HSCT. However, our study has certain limitations, which include—due to its retrospective design— missing data on alternative body composition analyses, such as body impedance analysis, as well as tests assessing muscle strength and quality of life. Still, we clearly link muscle quality – easily assessed by the IMAC in routine CT analyses – to adverse outcomes. Although it currently remains unclear whether and how muscle quality can be efficiently maintained or improved during AML treatment, identifying an easily assessable surrogate marker for sarcopenia marks a first important step towards an interventional study. Prospective analyses including nutritional and physical interventions are urgently needed to understand how muscle quality parameters, such as the IMAC, can be used to improve outcomes of AML patients undergoing HSCT.

## Supplementary information

Supplemental Material

## Data Availability

Data is available from the corresponding author upon reasonable request.
