# Supplementary material for: Low muscle quality, but not muscle mass, in abdominal CT-scans associates with adverse outcomes in AML patients undergoing an allogeneic stem cell transplantation
Source: Bone Marrow Transplant. 2026 Aug 13;61(11):1558–60. doi: 10.1038/s41409-026-03005-w (PMC13743492; doi:10.1038/s41409-026-03005-w)
Supplement: Supplementary file 1 [file 41409_2026_3005_MOESM1_ESM.docx]

**Supplementary Material to**

**Prognostic impact of sarcopenia in computed tomography in patients with acute myeloid leukemia undergoing allogeneic stem cell transplantation**

*Ussmann J, Wall C, et al.*

**Supplementary Methods**

Patient characteristics

We retrospectively analyzed 429 AML patients who underwent an allogeneic HSCT at our center between June 2001 and December 2022. All enrolled patients had received an abdominal computer tomography (CT) scan up to 7 weeks prior to allogeneic HSCT as part of the routine work-up to exclude an infectious focus. Median time from diagnosis to HSCT was 135 days. Remission status at allogeneic HSCT was first composite complete remission (CRc, 65%), later CRc (17%), or relapsed/refractory disease (18%). Conditioning regimens were myeloablative (25%), reduced intensity (15%), or non-myeloablative (60%). Further patients’ characteristics at diagnosis and allogeneic HSCT are given in Supplementary Table S1 and Table S3. Median follow up after allogeneic HSCT for patients alive was 4.2 years.

**Body Composition Analysis in CT scans**

CT scans covered the abdomen, and pelvis and were performed on a 128-slice or 256-slice CT scanner (Ingenuity or iCT256, Philips, Hamburg, Germany). An iodine-based contrast medium (90 mL Imeron 400 MCT, Bracco Imaging Germany GmbH, Konstanz, Germany) was given at a rate of 2-4 mL/s via a venous line (peripheral or central). An automatic bolus tracking in the descending aorta with a trigger of 100 Hounsfield units (HU) was used to obtain a CT in the portal-venous phase with the imaging parameters: 100 kVp; 125 mAs; slice thickness, 1 mm; pitch, 0.9.

One reader, blinded for clinical outcomes, measured the body composition parameters with the open-source software ImageJ 1.48v (National Institutes of Health Image program). A potential reader-bias was minimized by the semiautomatic standardized manner of selecting regions of interest (ROI).

Muscle mass and skeletal muscle index (SMI)

The ROI for skeletal muscle area quantification was determined using threshold levels of -29 and 150 Hounsfield Units (HU), as previously described,^1^ and included psoas, paraspinal and abdominal wall muscles on the L3 level (Figure S1A-B). To calculate the skeletal mass index (SMI), the skeletal muscle area was divided by the body height squared. To define low muscle mass, the SMI threshold proposed by Prado *et al.* was used (below 52.4 cm²/m² for males and below 38.5 cm²/m² for females).^2^ The SMI was evaluable for 425 patients due to missing data on height in four patients.

Definition of IMAC cut-off values

The IMAC was calculated by dividing the mean skeletal muscle density by the subcutaneous tissue density, as described by Hamaguchi *et al..*^3^ As no established cut-offs to define sarcopenia by a lower muscle quality (*i.e.* higher IMAC) in cancer patients exist, we defined informative cut-off values within our patient-cohort. First, the dataset was randomly partitioned by sex to ensure equal sex distribution and accommodate varying cut-off values, with one-third allocated to the test set and two-thirds to the confirmation set. The test and confirmation sets had similar patient characteristics (Table S3) and outcomes (Figure S6).

Using the R package ‘OptimalCutpoints’ in the test set, receiver operating characteristic (ROC) curves were used to determine cut-off values for the IMAC (-0.2469 for males and -0.2146 for females). These cut-off values were then validated in the confirmation set. The presented outcome and association analyses include the combined data of the test and confirmation set (for separate outcome analysis please see Figure S7).

Induction chemotherapy protocols

Prior to allogeneic HSCT, all patients received frontline therapy with either standard intensive Cytarabine-based cytarabine-based chemotherapy or hypomethylating agents with or without venetoclax. These included conventional 7+3 (n=66), CPX-351 (n=4)^4^ or treatment within or according to the OSHO studies (#061 or #069,^5,6^ under or over 60 years, n=308), the Ratify Trial (n=12),^7^ or the Quantum first trial (n=3).^8^ Additional 27 patients received Azacitidine followed by OSHO induction. Nine patients received non-intensive treatments with either Azacitidine alone (n=7) or in combination with Venetoclax (n=2).^9^

Conditioning regimens prior to allogeneic HSCT

Conditioning was either non-myeloablative (NMA) consisting of 3x30 mg/m^2^ Fludarabine and 2 Gy total body irradiation (TBI), myeloablative (MAC) consisting of either 2x60 mg/kg body weight Cyclophosphamide and 12 Gy TBI or 5x30 mg/m^2^ Fludarabine and 8 Gy TBI, or of reduced intensity (RIC) consisting of either busulfan (8 mg/kg orally or 6.4 mg/kg intravenously) or Treosulfan (3x10 g/m^2^) and 5x30 mg/m^2^ Fludarabine,^10^ Fludarabine and Melphalan,^11^ Fludarabine, Thiothepa, and Melphalan,^12^ or FLAMSA-based conditioning.^13^

Immunosuppression

In the majority of patients, graft-versus-host disease (GvHD) prophylaxis was started intravenously at a dose of 5 mg/kg body weight Cyclosporine A in two daily doses from day -1 which was adjusted to a whole-blood target level of 120-150 ng/ml for patients receiving FLAMSA conditioning or 200 ng/ml for all others.

Patients receiving NMA-HSCT additionally obtained Mycophenolate Mofetil 3 g per day in three daily doses in case of unrelated HSCT or 2 g per day in two daily doses in case of related HSCT. None of the patients undergoing NMA-HSCT received *in vivo* T-cell depletion.

When treated with FLAMSA conditioning, patients additionally received 2 g Mycophenolate Mofetil per day, which was stopped at day 28. Patients undergoing RIC and MAC-HSCT, received additional Methotrexate 15 mg administered intravenously on days +1, +3, +6, and +11 after HSCT, and RIC and MAC patients transplanted from an unrelated donor also received Thymoglobulin 2 mg/kg per day for three days as *in vivo* T-cell depletion. Reduction of Cyclosporine A started on day +42 and Cyclosporine A was stopped on day 120 following FLAMSA conditioning. For all others reduction of immunosuppressive therapy started on day +84 or day +180 following related or unrelated HSCT, respectively. Mycophenolate Mofetil was stopped at day +28 following related HSCT after NMA conditioning, and following unrelated HSCT tapered from days +40 to +96.^6^

Patients who underwent FLAMSA conditioning could receive up to three prophylactic donor lymphocyte infusions (DLI) per protocol after day 120 in increasing doses (5 x 10^5^/kg, 5 x 10^6^/kg, and 1 x 10^7^/kg recipient weight for patients receiving unrelated HSCT and 1 x 10^6^/kg, 5 x 10^6^/kg, and 1 x 10^7^/kg recipient weight for patients receiving related HSCT), if there was no evidence of GvHD.

Patients receiving haploidentical HSCT received *in vivo* T-cell depletion with Cyclophosphamide (50 mg/kg) at day +3 and +4 post-HSCT and as of day +5, immunosuppressive therapy with tacrolimus was started intravenously, initially administred continuously at a dose of 1mg/day, later orally twice daily, adjusted to a whole-blood target level of 5-15 ng/ml. Dose reduction started at day +180. At day +5, Mycophenolate Mofetil 3 g per day in three daily doses was started and stopped at day +35.^14^

Graft-versus-host disease

Evaluation for incidence of acute GvHD and chronic GvHD followed established criteria of the Glucksberg grading system.^15^ Requirement for acute GvHD was engraftment while engraftment and survival for at least 100 days after HSCT were required for chronic GvHD.

In cases of GvHD (grade > 2 according to Glucksberg grading system), Immunosuppression was prolonged or extended with systemic steroids.^15^

Definition of complete remission and active disease

According to ELN2022 classification, complete remission (CR) was defined as the presence of <5% blasts in bone marrow, neutrophils >1.0 x 10^9^/L, platelets >100 x 10^9^/L, absence of blasts with Auer rods in peripheral blood, independence of blood transfusion and no extramedullary disease.^16^ CR with incomplete count recovery (CRi) was defined as CR with platelets <100 x 10^9^/L or neutrophils <1.0 x 10^9^/L. The presence of CR or CRi was confirmed within 28 days prior to HSCT by bone marrow and peripheral blood analysis after HSCT. In case of persisting blast count >5% in bone marrow, persisting blasts in peripheral blood or the detection of extramedullary disease at HSCT disease status was defined as active disease. In this study, CR, CRi and morphologic leukemia-free state (MLFS) were summarized as composite CR (CRc).

Cytogenetics and Molecular Markers

Standard techniques of banding and *in situ* hybridization were used to perform cytogenetic analyses at diagnosis. Pretreatment genomic DNA was screened for the presence of *FLT3*-ITD, and the mutation status of the genes *CEBPA* and *NPM1* as previously described.^17,18^ In patients with adequate samples available, the diagnostic mutation status of recurrently mutated genes in myeloid malignancies was evaluated using next generation sequencing (Illumina, San Diego, CA, USA) as previously described.^19^ With the derived data, patients were grouped according to the ELN2022 genetic risk groups.

Statistical Analyses

The competing risks cumulative incidence of relapse (CIR) and NRM were calculated from allogeneic HSCT until relapse or death without relapse, respectively, using the Fine and Gray method.^20^ OS was calculated from allogeneic HSCT until death from any cause using the Kaplan-Meier method and groups were compared using the log-rank test. Associations with baseline clinical, genetic, and HSCT-related features were compared using the Kruskal-Wallis-Test and Fisher’s exact tests for continuous and categorical variables, respectively. Multivariate Analysis was performed as COX-regression models considering variables significant at α=0.10 in univariate analyses. All statistical analyses were performed using the R statistical software platform (version 4.0.2).^21^

Multivariate analyses

Variables considered in the multivariate models were those significant at α=0.10 in univariate analyses. Variables tested for multivariate analyses for all endpoints were patient sex, disease origin, ELN2022 genetic risk group, remission status at HSCT, conditioning regimen, donor type, IMAC prior to HSCT, SMI prior to HSCT, patient age at HSCT (< *vs.* >60 years), donor sex, CMV risk of patient and donor, and BMI at HSCT.

**Supplementary Results**

Patient characteristics and outcomes according to the SMI prior to allogeneic HSCT

In our analysis, nearly two-third of AML patients prior to allogeneic HSCT were sarcopenic defined by muscle mass. Patients with a lower muscle mass, *i.e.* an SMI below the published cut-off value,^2^ were more often male (*P*<.001), had a lower BMI at HSCT (*P*<.001) and a higher IMAC (*P*<.001), for detailed characteristics according to the SMI please see Table S4. However, CIR, NRM, and OS did not differ from that of patients with a higher SMI (Figure 2A,B), which was also true for separate analyses in female and male patients (Figure S3A,B). Similar results were observed when an optimal cut for the SMI (derived in the test set using ROC curve analyses similar to those described for IMAC) was used (Figure S5).

As for the intramuscular adipose tissue content (IMAC, see below), we also determined optimal cut-off values for SMI using ROC curves. Cut-off values were determined in the test-set and then validated in the confirmation-set. The optimal cut-off values were 52.36 cm²/m² for male patients and 40.38 cm²/m² for female patients. Also SMI devided by optimal cut-offs did not associate with outcomes in the test- or validation sets as well as in the whole cohort (Figure S5).

Patient characteristics according to the IMAC prior to allogeneic HSCT

Patients with a lower muscle quality (reflected by a higher IMAC) were older (*P*<.001), had a higher BMI at HSCT (*P*<.001), and a higher SMI (*P*<.001). They had a lower white blood cell count at diagnosis (*P*=.02), more often had a complex karyotype (*P*=.03), and were more frequently classified as European LeukemiaNet (ELN)-2022 adverse risk (*P*=.03). Regarding HSCT-related characteristics, patients with a high IMAC had a higher hematopoietic cell transplantation-specific comorbidity index (HCT-CI) risk score (*P*=.02), more often received reduced intensity or non-myeloablative conditioning (*P*<.001), and more often received allogeneic HSCT from an unrelated donor (*P*=.006) or mismatched donor (*P*=.006, see Table S1).

Prognostic relevance of the IMAC prior to allogeneic HSCT

Patients with a high IMAC were more likely to develop acute Graft-versus-Host Disease (GvHD) of grade two or higher (*P*=.001) and chronic GvHD (*P*=.04) compared to patients with a low IMAC. A high IMAC was associated with a higher NRM (*P*<.001) and shorter OS (*P*<.001), while CIR did not differ significantly (Figure 1A,B). These prognostic associations were also seen independently from patients’ sex (Figure S3).

In multivariate analyses, a high IMAC retained its prognostic significance, indicating a 3.57-times higher risk for NRM after adjustment for ELN2022 risk at diagnosis as well as a 2.17-times higher risk for shorter OS after adjustment for ELN2022 risk at diagnosis and the remission status at allogeneic HSCT (Table S2).

Prognostic relevance of the IMAC in distinct subgroups

Because of the strong associations of the IMAC with patient’s age (Figure 1C) as well as BMI (Figure 1E) at HSCT, subgroup analyses in different age and BMI subgroups were performed.

In patients aged ≤60 years at HSCT, a high IMAC showed strong associations with higher NRM (*P*<.001, cox-analysis Hazard Ratio [HR] 6.16, 95% Confidence Interval [CI] 2.59-14.6) and shorter OS (*P*<.001, cox-analysis HR 2.30, 95% CI 1.50-3.52), while weaker prognostic relevance was observed in patients >60 years at HSCT (NRM, *P*=.42, cox-analysis HR 1.38, 95% CI 0.62-3.19; and OS, *P*=.04, cox-analysis HR 1.81, 95% CI 1.01-3.22, Figure S4A-B).

Within the group of underweight and normal-weight patients (BMI ≤ 25kg/m^2^), a high IMAC was strongly associated with higher NRM (*P*<.001, cox-analysis HR 3.23 [1.61-6.48]) and shorter OS (*P*<.001, cox-analysis HR 2.48 [1.63-3.76]). In contrast, in overweight patients (BMI 25kg/m^2^-30kg/m^2^) higher IMAC associated with higher NRM (*P*=.04, cox-analysis HR 3.77 [0.93-15.30]), but only a trend towards shorter OS (*P*=.07, cox-analysis HR 1.81 [0.95-3.44]), and in obese patients (BMI > 30kg/m^2^) a high IMAC did not associate with outcomes (NRM, *P*=.26, cox-analysis HR 2.96 [0.40-22] and OS, *P*=.40, HR 1.69 [0.51-5.64], Figure S4C-D).

Finally, there also was an association between the IMAC and the development of an acute and chronic GvHD. Of note, when patients who did or did not develop a GVHD were regarded separately, the IMAC retained its prognostic relevance for OS in patients with or without an acute (*P*=.007 and *P*=.003, respectively) or chronic (*P*=.10 and *P*<.001, respectively) GvHD.

**References**

1.	Baggerman MR, van Dijk DPJ, Winkens B, et al. Muscle wasting associated co-morbidities, rather than sarcopenia are risk factors for hospital mortality in critical illness. *J Crit Care*. 2020;56. doi:10.1016/j.jcrc.2019.11.016

2.	Prado CM, Lieffers JR, McCargar LJ, et al. Prevalence and clinical implications of sarcopenic obesity in patients with solid tumours of the respiratory and gastrointestinal tracts: a population-based study. *Lancet Oncol*. 2008;9(7). doi:10.1016/S1470-2045(08)70153-0

3.	Hamaguchi Y, Kaido T, Okumura S, et al. Impact of quality as well as quantity of skeletal muscle on outcomes after liver transplantation. *Liver Transplantation*. 2014;20(11). doi:10.1002/lt.23970

4.	Lancet JE, Uy GL, Cortes JE, et al. Cpx-351 (cytarabine and daunorubicin) liposome for injection versus conventional cytarabine plus daunorubicin in older patients with newly diagnosed secondary acute myeloid leukemia. *Journal of Clinical Oncology*. 2018;36(26):2684-2692. doi:10.1200/JCO.2017.77.6112

5.	Büchner T, Schlenk RF, Schaich M, et al. Acute Myeloid Leukemia (AML): Different treatment strategies versus a common standard arm - Combined prospective analysis by the German AML Intergroup. *Journal of Clinical Oncology*. 2012;30(29):3604-3610. doi:10.1200/JCO.2012.42.2907

6.	Niederwieser D, Hoffmann VS, Pfirrmann M, et al. Comparison of Treatment Strategies in Patients over 60 Years with AML: Final Analysis of a Prospective Randomized German AML Intergroup Study. [abstract]. In: *Blood*. Vol 128. 2016:1066.

7.	Stone RM, Mandrekar SJ, Sanford BL, et al. Midostaurin plus chemotherapy for acute myeloid leukemia with a FLT3 Mutation. *New England Journal of Medicine*. 2017;377(5):454-464. doi:10.1056/NEJMoa1614359

8.	Erba HP, Montesinos P, Kim HJ, et al. Quizartinib plus chemotherapy in newly diagnosed patients with FLT3-internal-tandem-duplication-positive acute myeloid leukaemia (QuANTUM-First): a randomised, double-blind, placebo-controlled, phase 3 trial. *The Lancet*. 2023;401(10388). doi:10.1016/S0140-6736(23)00464-6

9.	DiNardo CD, Jonas BA, Pullarkat V, et al. Azacitidine and Venetoclax in Previously Untreated Acute Myeloid Leukemia. *New England Journal of Medicine*. 2020;383:617-629. doi:10.1056/NEJMoa2012971

10.	Kröger N, Iacobelli S, Franke GN, et al. Dose-reduced versus standard conditioning followed by allogeneic stem-cell transplantation for patients with myelodysplastic syndrome: A prospective randomized phase III study of the EBMT (RICMAC Trial). *Journal of Clinical Oncology*. 2017;35(19):2157-2164. doi:10.1200/JCO.2016.70.7349

11.	Bryant A, Nivison-Smith I, Pillai ES, et al. Fludarabine Melphalan reduced-intensity conditioning allotransplanation provides similar disease control in lymphoid and myeloid malignancies: Analysis of 344 patients. *Bone Marrow Transplant*. 2014;49(1):17-23. doi:10.1038/bmt.2013.142

12.	Duque-Afonso J, Ihorst G, Waterhouse M, et al. Comparison of reduced-toxicity conditioning protocols using fludarabine, melphalan combined with thiotepa or carmustine in allogeneic hematopoietic cell transplantation. *Bone Marrow Transplant*. Published online 2020:1-11. doi:10.1038/s41409-020-0986-2

13.	Pfrepper C, Klink A, Behre G, et al. Risk factors for outcome in refractory acute myeloid leukemia patients treated with a combination of fludarabine, cytarabine, and amsacrine followed by a reduced-intensity conditioning and allogeneic stem cell transplantation. *J Cancer Res Clin Oncol*. 2016;142(1):317-324. doi:10.1007/s00432-015-2050-y

14.	Luznik L, O’Donnell P V., Symons HJ, et al. HLA-Haploidentical Bone Marrow Transplantation for Hematologic Malignancies Using Nonmyeloablative Conditioning and High-Dose, Posttransplantation Cyclophosphamide. *Biology of Blood and Marrow Transplantation*. 2008;14(6). doi:10.1016/j.bbmt.2008.03.005

15.	Glucksberg H, Storb R, Fefer A, et al. Clinical manifestations of graft-versus-host disease in human recipients of marrow from HL-A-matched sibling donors. Preprint posted online 1974:295-304.

16.	Döhner H, Estey EH, Amadori S, et al. Diagnosis and management of acute myeloid leukemia in adults: Recommendations from an international expert panel, on behalf of the European LeukemiaNet. *Blood*. 2010;115(3):453-474. doi:10.1182/blood-2009-07-235358

17.	Jentzsch M, Bill M, Grimm J, et al. High expression of the stem cell marker GPR56 at diagnosis identifies acute myeloid leukemia patients at higher relapse risk after allogeneic stem cell transplantation with the CD34+/CD38- population. *Haematologica*. 2020;105(10):e507.

18.	Grimm J, Jentzsch M, Bill M, et al. Prognostic impact of the ELN2017 risk classi fi cation in patients with AML receiving allogeneic transplantation. *Blood Adv*. 2020;4(16):3864-3874. doi:10.1182/bloodadvances.2020001904

19.	Grimm J, Bill M, Jentzsch M, et al. Clinical impact of clonal hematopoiesis in acute myeloid leukemia patients receiving allogeneic transplantation. *Bone Marrow Transplant*. 2019;54(8). doi:10.1038/s41409-018-0413-0

20.	Fine JP, Gray RJ. A Proportional Hazards Model for the Subdistribution of a Competing Risk. *J Am Stat Assoc*. 1999;94(446). doi:10.1080/01621459.1999.10474144

21.	R Core Team. R Core Team 2021 R: A language and environment for statistical computing. R foundation for statistical computing. https://www.R-project.org/. *R Foundation for Statistical Computing*. 2022;2.

**Supplementary Tables**

**Supplementary Table S1:** Patients’ characteristics according to the IMAC prior to allogeneic HSCT (n=429)

|  | **All patients**  **n=429** | **Low IMAC**  **n=138** | **High IMAC**  **n=291** | ***P*** |
| --- | --- | --- | --- | --- |
| Characteristics at diagnosis |  |  |  |  |
| Sex, n (%)  male  female | 230 (54)  199 (46) | 72 (52)  66 (48) | 158 (54)  133 (46) | .76 |
| Disease origin, n (%)  secondary/treatment related  *de novo* | 153 (36)  276 (64) | 37 (27)  101 (73) | 116 (40)  175 (60) | **.01** |
| Hemoglobin, g/dL  median (range) | 8.9 (3.8-15.3) | 8.9 (4.2-15) | 8.9 (3.8-15.3) | .78 |
| Platelet count, x 10^9^/L  median (range) | 62 (1.6-950) | 69.5 (5-517) | 54 (1.6-950) | .11 |
| WBC, x 10^9^/L  median (range) | 6.5 (0.6-432) | 12.9 (0.6-432) | 5.4 (0.7-385) | **.02** |
| Blood blasts, %  median (range) | 20 (0-97) | 23 (0-96) | 20 (0-97) | .92 |
| Bone marrow blasts, %  median (range) | 52 (0-100) | 55 (0-94) | 51 (0-100) | .81 |
| LDH, ukat/l  median (range) | 6.0 (1.5-36.3) | 6.9 (1.5-36.3) | 5.7 (2.5-32.2) | **.05** |
| RDW, %  median (range) | 16.7 (13-30.6) | 16.6 (13.3-30.6) | 16.8 (13-29.6) | .97 |
| Complex karyotype*, n (%)  absent  present | 320 (75)  66 (15) | 113 (82)  14 (10) | 207 (71)  52 (18) | **.02** |
| ELN2022 risk  favorable  intermediate  adverse | 62 (14)  77 (18)  147 (34) | 21 (22)  34 (36)  39 (41) | 41 (21)  43 (22)  108 (56) | **.03** |
| Characteristics at HSCT |  |  |  |  |
| Age at HSCT, years  median (range) | 59.9 (17.5-76.4) | 47.8 (19.3-76.4) | 63.1 (17.5-74.9) | **<.001** |
| Time from diagnosis to HSCT (d)  median (range) | 135 (23-1316) | 130 (41-996) | 139 (23-1316) | **.04** |
| Remission status at HSCT, n (%)  first CR/CRi  later CR/CRi  PR/relapsed/refractory | 280 (65)  73 (17)  76 (18) | 94 (68)  16 (12)  28 (20) | 186 (64)  57 (20)  48 (16) | .09 |
| Conditioning regimen, n (%)  MAC  RIC  NMA | 106 (25)  65 (15)  258 (60) | 68 (49)  24 (17)  46 (33) | 38 (13)  41 (14)  212 (73) | **<.001** |
| HCT-CI Score, n (%)  0  1/2  ≥ 3 | 176 (42)  109 (26)  133 (32) | 68 (51)  35 (26)  31 (23) | 108 (38)  74 (26)  102 (36) | **.02** |
| BMI at HSCT, n (%)  underweight (<18.5)  normal (18.5 – < 25)  overweight (25 – < 30)  obese (>= 30) | 13 (3)  218 (52)  138 (33)  47 (11) | 7 (5)  89 (67)  29 (22)  7 (5) | 6 (2)  129 (45)  109 (38)  40 (14) | **<.001** |
| SMI at HSCT, n (%)  median (range) | 42.8 (20.4-70.1) | 40.1 (20.4-60.1) | 43.6 (25.4-70.1) | **<.001** |
| Donor type, n (%)  matched related  unrelated, HLA matched  HLA mismatched  haploidentical | 73 (17)  266 (62)  82 19)  6 (1) | 34 (25)  86 (62)  16 (12)  2 (1) | 39 (13)  180 (62)  66 (23)  4 (1) | **.04** |
| Donor sex*,* n (%)  female into male  all others | 63 (15)  363 (85) | 21 (15)  116 (86) | 42 (15)  247 (85) | .88 |
| CMV status, n (%)  recipient + / donor –  all others | 159 (37)  267(63) | 46 (34)  91 (66) | 113 (39)  176 (61) | .29 |
| Outcomes after HSCT |  |  |  |  |
| Acute GvHD ≥ grade 2, n (%)  absent  present | 270 (72)  106 (28) | 99 (83)  21 (18) | 171 (67)  85 (33) | **.001** |
| Chronic GvHD, n (%)  absent  limited  extended | 119 (42)  39 (14)  123 (44) | 49 (51)  16 (17)  31 (32) | 70 (38)  23 (12)  92 (50) | **.02** |

*Abbreviations: CMV, cytomegalovirus; CR, complete remission; CRi, complete remission with incomplete peripheral recovery; d, days; ELN, European Leukemia Net; FLT3, fms-like tyrosine kinase; GvHD, graft-versus-host disease; Hb, hemoglobin; HLA, human leukocyte antigen; HCT-CI, hematopoietic cell transplantation comorbidity index; HSCT, hematopoietic stem cell transplantation; IMAC, intramuscular adipose tissue content; LDH, lactate dehydrogenase; MRD, measurable residual disease; PB, peripheral blood; PR, partial remission; RDW, red cell distribution width; SMI, skeletal muscle mass index; WBC, white blood count; WHO, World Health Organization.*

**complex karyotype according to ELN2022*

**Supplemetary Table S2:** Multivariate analysis.

|  | Non-relapse mortality | | Overall survival | |
| --- | --- | --- | --- | --- |
|  | **HR* (95% CI)** | ***P*** | **OR** (95% CI)** | ***P*** |
| ELN2022 genetic risk  (adverse *vs* intermediate *vs* favorable) | 0.66 (0.49 – 0.89) | .006 | 0.77 (0.63 – 0.95) | .02 |
| Remission status at HSCT  (first CR/CRi *vs* later CR/CRi *vs* no CR/CRi) | - | - | 0.70 (0.56 - 0.86) | <.001 |
| IMAC prior to HSCT  (high *vs* low, optimal cut) | 3.57 (1.76 – 7.27) | <.001 | 0.46 (0.31 – 0.68) | <.001 |

*Abbreviations: CI, confidence interval; CR, complete remission; CRi, CR with incomplete peripheral recovery; ELN2022, European LeukemiaNet 2022; HSCT, hematopoietic stem cell transplantation; IMAC, intramuscular adipose tissue content (optimal cut: males -0.2469; females -0.2146).*

**HR, hazard ratio, <1 (>1) indicate lower (higher) risk of relapse/non-relapse mortality for the first category listed for the dichotomous variables for the lower (higher) values of the continuous variables.*

***OR, odds ratio, <1 (>1) indicate lower (higher) chance of survival for the first category listed for the dichotomous variables.*

*For NRM endpoint, variables considered were: disease origin (de novo vs secondary or treatment related), ELN2022 genetic risk group, conditioning regimen (non-myeloablative vs reduced intensity vs myeloablative), donor type (matched related vs matched unrelated vs mismatched), IMAC prior to HSCT (high vs low, optimal cut), and age at HSCT (< vs >60 years).*

*For OS endpoint, variables considered were: disease origin (de novo vs secondary or treatment related), ELN2022 genetic risk group, remission at HSCT (first CR/CRi vs later CR/CRi vs no CR/CRi ), conditioning regimen (non-myeloablative vs reduced intensity vs myeloablative), IMAC prior to HSCT (high vs low, optimal cut), and age at HSCT.*

**Supplementary Table S3:** Patient’s characteristics within the test set and confirmation set.

|  | **Test set**  **n=142** | **Validation set**  **n=287** | ***P*** |
| --- | --- | --- | --- |
| Characteristics at diagnosis |  |  |  |
| Sex, n (%)  male  female | 76 (54)  66 (46) | 154 (54)  133 (46) | >.99 |
| Disease origin, n (%)  secondary/treatment related  *de novo* | 51 (35)  91 (64) | 102 (36)  185 (64) | >.99 |
| Hemoglobin, g/dL  median (range) | 8.9 (4,5-13,4) | 9 (3.8-15.3) | .67 |
| Platelet count, x 10^9^/L  median (range) | 63 (2-488) | 62 (1.6-950) | .51 |
| WBC, x 10^9^/L  median (range) | 5.1 (0,6-385) | 8.4 (0,7-432) | .55 |
| Blood blasts, %  median (range) | 25 (0-97) | 18 (0-97) | .36 |
| Bone marrow blasts, %  median (range) | 52 (14.95) | 52 (0-100) | .33 |
| CD34+/CD38- cell burden  median (range) | 0.7 (0-68) | 0.5 (0-89) | .34 |
| LDH, ukat/l  median (range) | 5.41 (2.9-30) | 6.16 (1.5-36.3) | .40 |
| RDW, %  median (range) | 16.8 (13-26.8) | 16.6 (13.1-30.6) | .43 |
| Normal karyotype, n (%)  absent  present | 81 (61)  52 (39) | 159 (59)  110 (41) | .75 |
| ELN 2022 risk  favorable  intermediate  adverse | 23 (23)  31 (31)  47 (47) | 39 (21)  46 (25)  100 (54) | .43 |
| Characteristics at HSCT |  |  |  |
| Age at HSCT, years  median (range) | 57 (20-75) | 60 (17-76) | .58 |
| Remission status at HSCT, n (%)  CR/CRi  CR, MRD^neg^  CR, MRD^pos^  CR, no MRD available  PR/relapsed/refractory | 116 (82)  42 (46)  24 (26)  50 (35)  26 (28) | 239 (83)  80 (44)  54 (30)  105 (37)  47 (26) | .81 |
| Conditioning regimen, n (%)  NMA  RIC  MAC | 89 (63)  18 (13)  35 (25) | 169 (59)  47 (16)  71 (25) | .59 |
| HCT-CI Score, n (%)  0  1/2  ≥ 3 | 57 (42)  41 (30)  39 (28) | 119 (43)  68 (25)  87 (32) | .54 |
| BMI at HSCT, n (%)  underweight (<18.5)  normal (18.5 – < 25)  overweight (25 – < 30).  obese (>= 30) | 5 (4)  70 (52)  42 (31)  28 (13) | 8 (3)  148  96 (34)  29 (10) | .73 |
| SMI at HSCT  median (range) | 42.9 (25.1 – 64.4) | 42.8 (20.4–70.1) | .89 |
| IMAC at HSCT  median (range) | -0.199 (-5.324 – 0.042) | -0.191 (-212.447 – 16.782) | .58 |
| Donor type, n (%)  matched related  unrelated, HLA matched  HLA mismatched  haploidentical | 17 (12)  93 (66)  29 (21)  2 (1) | 56 (20)  173 (60)  53 (19)  4 (1) | .26 |
| Donor sex*,* n (%)  female into male  all others | 18 (13)  122 (87) | 45 (16)  241 (84) | .47 |
| CMV status, n (%)  recipient + / donor –  all others | 53 (38)  86 (62) | 106 (37)  181 (63) | .75 |
| Outcomes after HSCT |  |  |  |
| Relapse after HSCT, n (%)  yes  no | 53 (37)  89 (63) | 117 (41)  170 (59) | .53 |
| Death after HSCT, n (%)  yes  no | 64 (45)  78 (55) | 153 (53)  134 (47) | .12 |
| Acute GvHD ≥ grade 2, n (%)  absent  present | 88 (73)  33 (27) | 182 (71)  73 (29) | .81 |
| Chronic GvHD, n (%)  absent  limited  extended | 41 (43)  13 (14)  41 (43) | 78 (42)  26 (14)  82 (44) | .98 |

*Abbreviations: CMV, cytomegalovirus; CR, complete remission; CRi, complete remission with incomplete peripheral recovery; ELN, European Leukemia Net; FLT3, fms-like tyrosine kinase; GvHD, graft-versus-host disease; Hb, hemoglobin; HLA, human leukocyte antigen; HCT-CI, hematopietic cell transplantation comorbidity index; HSCT, hematopoietic stem cell transplantation; IMAC, intramuscular adipose tissue content (optimal cut: males -0.2469; females -0.2146); LDH, lactatedehydrogenase; MRD, measurable residual disease; PB, peripheral blood; PR, partial remission; RDW, red cell distribution width; WBC, white blood count; WHO, World Health Organization.*

**Supplementary Table S4 – Patient’s characteristica according to SMI**

|  | **SMI above cut-off***  **n=147** | **SMI below cut-off***  **n=278** | ***P*** |
| --- | --- | --- | --- |
| Characteristics at diagnosis |  |  |  |
| Sex, n (%)  male  female | 50 (34)  97 (66) | 178 (64)  100 (35) | **<.001** |
| Disease origin, n (%)  secondary/treatment related  *de novo* | 46 (31)  101 (69) | 105 (38)  173 (62) | .21 |
| ELN 2022 risk  favorable  intermediate  adverse | 25 (25)  26 (26)  51(50) | 36 (20)  51 (28)  95 (52) | .64 |
| Characteristics at HSCT |  |  |  |
| Age at HSCT, years  median (range) | 60.28 (21.87-74.75) | 59.6 (17.46-76.39) | .34 |
| Remission status at HSCT, n (%)  CR/CRi  PR/relapsed/refractory | 121 (82)  26 (18) | 231 (83)  47 (17) | .89 |
| Conditioning regimen, n (%)  NMA  RIC  MAC | 96 (65)  23 (16)  28 (19) | 158 (108)  42 (29)  78 (53) | .12 |
| HCT-CI Score, n (%)  0  1/2  ≥ 3 | 64 (44)  37 (26)  44 (30) | 111 (41)  71 (26)  89 (33) | .79 |
| BMI at HSCT, n (%)  underweight (<18.5)  normal (18.5 – < 25)  overweight (25 – < 30)  obese (>= 30) | 0 (0)  37 (26)  64 (45)  42 (29) | 13 (5)  181 (66)  74 (27)  5 (2) | **<.001** |
| IMAC at HSCT  median (range) | -0.171 (-0.672 – 0.084) | -0,206 (-212.447 – 16.782) | **<.001** |
| Donor type, n (%)  matched related  unrelated, HLA matched  HLA mismatched  haploidentical | 22 (15)  89 (61)  31(21)  4 (3) | 49 (18)  177 (64)  49 (18)  2 (1) | .28 |
| Donor sex*,* n (%)  female into male  all others | 18 (12)  127 (88) | 45 (16)  232 (84) | .32 |

***Cut-off for SMI according to Prado et al. ^2^**

*Abbreviations: CMV, cytomegalovirus; CR, complete remission; CRi, complete remission with incomplete peripheral recovery; ELN, European Leukemia Net; FLT3, fms-like tyrosine kinase; GvHD, graft-versus-host disease; Hb, hemoglobin; HLA, human leukocyte antigen; HCT-CI, hematopietic cell transplantation comorbidity index; HSCT, hematopoietic stem cell transplantation; IMAC, intramuscular adipose tissue content (optimal cut: males -0.2469; females -0.2146); LDH, lactatedehydrogenase; MRD, measurable residual disease; PB, peripheral blood; PR, partial remission; RDW, red cell distribution width; WBC, white blood count; WHO, World Health Organization.*

**Supplementary Figures**

**Figure S1**

**Figure S1. Representation of the performed measurements in CT scans. A-B** Selection of the skeletal muscle area in **(A)** a patient with a skeletal muscle index (SMI) above cut-off value, *i.e.* higher muscle mass and **(B)** a patient with an SMI below cut-off value, *i.e.* lower muscle mass. **C-D** Determination of Hounsfield units of skeletal muscle and subcutaneous adipose tissue in **(C)** a patient with an intramuscular adipose tissue content (IMAC) below cut-off value, *i.e.* higher muscle quality and **(D)** a patient with an IMAC above cut-off value *i.e.* lower muscle quality.

**Figure S2**

**Figure S2. Outcomes of AML patients undergoing allogeneic HSCT according to muscle mass defined by SMI. (A)** Competing risk analysis (Cumulative incidence of relapse/progression and non-relapse mortality) and **(B)** Overall survival according to the cut-off values (52.4 cm²/m² for males and 38.5 cm²/m² for females) initially published by Prado *et al*.^2^

**Figure S3**

**Figure S3.** **Outcomes of AML patients undergoing allogeneic HSCT according to patient-sex and muscle quality defined by the IMAC prior to HSCT (high *vs* low, optimal cut: males -0.2469; females -0.2146). (A)** Overall survival of male patients and **(B)** Overall survival of female patients.

**Figure S4**

**Figure S4. Outcomes of AML patients undergoing allogeneic HSCT according to age or BMI and muscle quality defined by the IMAC prior to HSCT (high *vs* low, optimal cut: males -0.2469; females -0.2146). (A)** Non-relapse mortality and **(B)** Overall survival in patients aged >60 years and **(C)** Non-relapse mortality and **(D)** Overall survival in patients with a BMI >30 kg/m^2^ at HSCT.

**Figure S5**

**Outcomes of AML patients undergoing allogeneic HSCT according to their sex and muscle mass prior to HSCT using an optimal cutpoint approach for skeletal muscle index (SMI). (A-C)** Overall survival in the test set: **(A)** all patients, **(B)** male patients (SMI cut = 52.36cm²/m²), and **(C)** female patients (SMI cut = 40.38cm²/m²). **(D-F)** Overall survival in the confirmation set: **(D)** all patients, **(E)** male patients (SMI cut = 52.36), and **(F)** female patients (SMI cut = 40.38cm²/m²).

**Figure S6**

**Figure S6. Outcomes of AML patients undergoing allogeneic HSCT according to their allocation into the test- and confirmation set (n=429).** Competing risk analysis (Cumulative incidence of relapse/progression and Non-relapse mortality) and **(B)** Overall survival.

**Figure S7**

**Figure S7. Outcomes of AML patients undergoing allogeneic HSCT according to the IMAC prior to HSCT using an optimal cutpoint approach. (A)** Overall survival in male patients (optimal cut = -0.2469), and **(B)** Overall survival female patients (optimal cut = -0.2146) in the test set. **(C)** Overall survival in male patients (optimal cut = -0.2469), and **(D)** Overall survival female patients (-0.2146) in the confirmation set.
